# *TP53* Mutations in Serum Circulating Cell-Free Tumor DNA As Longitudinal Biomarker for High-Grade Serous Ovarian Cancer

**DOI:** 10.3390/biom10030415

**Published:** 2020-03-07

**Authors:** Silvia R. Vitale, Floris H. Groenendijk, Ronald van Marion, Corine M. Beaufort, Jean C. Helmijr, Hendrikus Jan Dubbink, Winand N. M. Dinjens, Patricia C. Ewing-Graham, Ramon Smolders, Helena C. van Doorn, Ingrid A. Boere, Els M. J. J. Berns, Jozien Helleman, Maurice P. H. M. Jansen

**Affiliations:** 1 Department of Medical Oncology, Erasmus MC Cancer Institute, Erasmus University Medical Center, 3015 CN Rotterdam, The Netherlands; c.beaufort@erasmusmc.nl (C.M.B.); j.helmijr@erasmusmc.nl (J.C.H.); i.boere@erasmusmc.nl (I.A.B.); petronellaberns@gmail.com (E.M.J.J.B.); j.helleman@erasmusmc.nl (J.H.); m.p.h.m.jansen@erasmusmc.nl (M.P.H.M.J.); 2 Department of Clinical and Experimental Medicine, University of Catania, 95123 Catania, Italy; 3 Center of Experimental Oncology and Hematology, A.O.U. Policlinico-Vittorio Emanuele, 95123 Catania, Italy; 4 Department of Pathology, Erasmus MC Cancer Institute, Erasmus University Medical Center, 3015 CN Rotterdam, The Netherlands; f.groenendijk@erasmusmc.nl (F.H.G.); r.vanmarion@erasmusmc.nl (R.v.M.); h.dubbink@erasmusmc.nl (H.J.D.); w.dinjens@erasmusmc.nl (W.N.M.D.); p.ewing@erasmusmc.nl (P.C.E.-G.); 5 Department of Gynaecology, Erasmus MC Cancer Institute, Erasmus University Medical Center, 3015 CN Rotterdam, The Netherlands; r.smolders@erasmusmc.nl (R.S.); h.vandoorn@erasmusmc.nl (H.C.v.D.)

**Keywords:** ovarian cancer, *TP53*, cell-free DNA, serum, dPCR, next-generation sequencing, molecular barcoding

## Abstract

The aim of this study was to determine an optimal workflow to detect *TP53* mutations in baseline and longitudinal serum cell free DNA (cfDNA) from high-grade serous ovarian carcinomas (HGSOC) patients and to define whether *TP53* mutations are suitable as biomarker for disease. *TP53* was investigated in tissue and archived serum from 20 HGSOC patients by a next-generation sequencing (NGS) workflow alone or combined with digital PCR (dPCR). AmpliSeq™-focused NGS panels and customized dPCR assays were used for tissue DNA and longitudinal cfDNAs, and Oncomine NGS panel with molecular barcoding was used for baseline cfDNAs. *TP53* missense mutations were observed in 17 tissue specimens and in baseline cfDNA for 4/8 patients by AmpliSeq, 6/9 patients by Oncomine, and 4/6 patients by dPCR. Mutations in cfDNA were detected in 4/6 patients with residual disease and 3/4 patients with disease progression within six months, compared to 5/11 patients with no residual disease and 6/13 patients with progression after six months. Finally, mutations were detected at progression in 5/6 patients, but not during chemotherapy. NGS with molecular barcoding and dPCR were most optimal workflows to detect TP53 mutations in baseline and longitudinal serum cfDNA, respectively. *TP53* mutations were undetectable in cfDNA during treatment but re-appeared at disease progression, illustrating its promise as a biomarker for disease monitoring.

## 1. Introduction

Epithelial ovarian cancer is the most lethal malignancy among gynecological cancers in the Western world, partly due to the advanced disease stage at the time of diagnosis in most patients [1]. A large proportion of these patients will have a recurrence or progression within two years and ultimately die of their disease. Only 30% of women initially diagnosed with advanced-stage disease will survive more than five years. However, when the disease is diagnosed while still confined to the ovary, five-year survival is 70% to 90% [2].

Circulating tumor DNA (ctDNA) and cell-free DNA (cfDNA) isolated from blood have recently been extensively investigated as potential blood-based biomarkers for several cancer types [3,4,5]. In ovarian cancer patients, higher levels of cfDNA were found as compared to healthy donors [6,7] and patients with benign disease [1]. Higher levels of cfDNA were associated with advanced disease stage, high grade, and poorer prognosis [1,8]. In addition, it has been reported in an orthotopic mouse model that progression of disease could be monitored by measuring human cfDNA. In this model, the amount of cfDNA correlated significantly with tumor weight [9]. However, increased level of cfDNA can also be found in patients with benign lesions, inflammatory disease, and tissue trauma and is therefore not a specific tumor biomarker [10]. The presence of tumor-specific genetic alterations in cfDNA could potentially offer a more specific approach.

In this context, it is of interest that 96% of high-grade serous ovarian carcinomas (HGSOC), representing the majority of advanced stage ovarian cancers, have a mutated *TP53* gene [11,12]. Therefore, the presence of *TP53* mutations in cfDNA could potentially be used as a tumor-specific marker for HGSOC. However, *TP53* mutations can also be detected in plasma DNA from healthy, especially older, individuals and patients with other tumor types [13]. We hypothesized that tumor-specific *TP53* missense mutations in archived serum cfDNA of HGSOC patients could be used as a biomarker at baseline, and as a marker to monitor tumor load during therapy and thereafter. This hypothesis is supported by data from a retrospective study of 40 HGSOC patients, which showed that plasma circulating tumor DNA correlated with disease load at start of therapy. Furthermore, a decrease of less than 60% in *TP53* mutation frequency after one cycle of chemotherapy was associated with shorter time to progression (TTP) [14]. However, to date, it is not clear which technique is optimal for detecting and monitoring *TP53* mutations in cfDNA.

In the current study, we evaluate different NGS workflows, used alone and combined with digital PCR (dPCR), for detecting *TP53* mutations in minute amounts of archived serum (<1 mL). These samples were taken at the time of diagnosis from 20 patients with advanced HGSOC patients. The amount of baseline cfDNA with *TP53* mutation was correlated with residual disease after debulking surgery and progression-free survival after platin-based chemotherapy. We also analyzed mutations in serum taken during chemotherapy and at disease progression in the same group of patients to investigate the potential of *TP53* mutations in blood as a marker of disease progression.

## 2. Materials and Methods

### 2.1. Study Design and Patient Characteristics

For this retrospective study, we selected 20 patients with FIGO stage IIIC–IV ovarian cancer diagnosed at the Erasmus MC Cancer Institute in Rotterdam, The Netherlands. This retrospective study of archived tissue and serum was approved by the medical ethics committee of the Erasmus MC Rotterdam, the Netherlands (MEC-2002-949; MEC-2008-183) and performed in accordance with the principles of the Declaration of Helsinki and the local law. The study was carried out according the REMARK guidelines and Code of Conduct of the Federation of Medical Scientific Societies in the Netherlands (https://www.federa.org/codes-conduct). The majority of patients had high-grade serous ovarian cancer (N = 19), only one with adenocarcinoma (N = 1). Most patients received primary or interval debulking surgery, and all received platinum-based chemotherapy. Patient subsets were defined based on size of residual disease (optimal debulking defined as 0–1 cm vs. non-optimal debulking defined as >1 cm residual disease) and progression-free survival (PFS ≤ 6 months vs. PFS > 6 months). The study evaluated two workflows (Table 1): Workflow I evaluated only *TP53* by NGS and dPCR on tissue DNA and longitudinal serum cfDNAs, whereas workflow II applied only NGS using multigene panels including *TP53* on tissue DNA and baseline cfDNA. Patient, clinical characteristics, and workflow details are summarized in Table 1 and Table 2, respectively.

### 2.2. DNA Isolation

The DNA was extracted from fresh frozen tumor tissue specimens (N = 8) and from formalin-fixed paraffin embedded (FFPE) tissue (N = 12) taken at debulking surgery as described previously [15]. Tissues were sectioned for DNA isolation and the percentage of tumor cells was evaluated in a hematoxylin-eosin-stained section as described earlier [16]. The cfDNA was isolated from archived minute amounts of serum samples taken at diagnosis for all patients in both workflows, and at chemotherapy and at disease progression for patients of workflow I. The QIAamp Circulating Nucleic Acid kit (Qiagen, KJ Venlo, The Netherlands) was used to isolate cfDNA from a median of 400 µL serum (range 100–1000 µL) according to the manufacturer’s manual. This cfDNA was isolated into 20 µL elution buffer. A Qubit^®^ 2.0 fluorimeter (Thermo Scientific, Carlsbad, California, USA) and the Quant-iT dsDNA high-sensitivity assay (Invitrogen, Life Technologies, Carlsbad, CA, USA) were used to quantify the isolated DNA yields and concentrations.

### 2.3. Next-Generation Sequencing

Different NGS panels and sequencer platforms were used in the two workflows all purchased from Life Technologies (Carlsbad, CA, USA). The panels differ in amplicon and target size, methodology, and sequencing costs per sample (Table 2). Tissue DNA was sequenced on the Ion Torrent Personal Genome Machine (ion-PGM) using two Ion Ampliseq focused panels. In workflow I, the Ion AmpliSeq™ TP53 community panel (24 amplicons, 2550 bp; analyzing exons and UTRs of the TP53 gene) was used. In workflow II, a customized Ion Ampliseq Diagnostic V5.1 targeted panel (328 amplicons, 35,793 bp, 41 genes) was applied to sequence all TP53 exons. Serum cfDNA was sequenced with the Ion AmpliSeq™ TP53 community panel on the ion-PGM (workflow I) or with the Oncomine breast cfDNA NGS assay with molecular barcoding (26 amplicons, 4420 bp, 10 genes) on an Ion S5XL sequencer (workflow II). The ampliseq panels have a limit of detection (LOD) for mutation frequencies of at least 1%, whereas the Oncomine panel has a LOD of 0.1% at 20 ng DNA input. For this lower LOD, much deeper read depth coverage is needed for the Oncomine panel (>20k coverage) than Ampliseq panels (<1k coverage). Consumables, kits, software packages, and protocols for the NGS analyses with Ampliseq and Oncomine focused panels were used as indicated by the manufacturer and as previously described by us [3,16,17]. Briefly, 10 ng tumor DNA was used for all patients as input in the Ampliseq library preparation. For cfDNA library preparations, at least 1.5 ng cfDNA was used with equal amounts for all three sera per patient for the Ampliseq panel in workflow I (median 2.2 ng; range per patient: 1.5–3.3 ng) and at least 15 ng up to 20 ng of cfDNA was used for the Oncomine panel in workflow II. Samples were sequenced on Ion 318 and 530 chips for workflows I and II, respectively. Sequencing with Ampliseq panels was performed with on average tumor tissue DNA reads depth coverage of 1725 reads/amplicon (range: 943–3584 reads) for workflow I and 1166 reads/amplicon (range: 430–1796 reads) for workflow II. The average cfDNA reads depth coverage was 1851 reads/amplicon (range: 843–3776 reads) for workflow I and 32,902 reads/amplicon (range: 5786–59,976 reads) for workflow II.

### 2.4. Bio-Informatics for SNV Detection and Evaluation

The Torrent Suite v4.0 (Thermo Scientific, Carlsbad, CA, USA) was used for raw data analyses, base calling, and alignment. Variant Caller v4.4.2.1 (VC, Thermo Scientific, Carlsbad, CA, USA) was applied to detect DNA sequence alterations. Annotation of the variants was performed by a custom pipeline including ANNOVAR (openbioinformatics.org/annovar) in a Galaxy (galaxyproject.org) environment. For the initial VC analysis of each tumor and serum DNA-sample, somatic low-stringency filter settings were applied to detect DNA variants when compared to the reference genome (hg19; build 37). For each sample, only sequences with 100 reads depth or more were evaluated. Subsequently, TP53 mutations were visually examined using Integrative Genomics Viewer (IGV, CA, USA) software (http//www.broadinstitute.org/igv).

### 2.5. Digital PCR

Independent validation of six identified TP53 mutations were performed using TaqMan^®^ SNP genotyping assays on the QuantStudio™ 3D Digital PCR system (Thermo Fisher Scientific, Waltham, MA, USA), according to the manufacturer specifications. All the assays, except TP53 p.R282W, were designed in-house using the Thermo Fisher Custom TaqMan^®^ Assay Design Tool and ordered as Custom TaqMan^®^ SNP Genotyping assay from Thermo Fisher (Supplementary Tables S1 and S2). The PCR reaction mix was prepared in a final volume of 17.4 µL containing 30 ng of tumor DNA or ranging from 3.6 ng to 20 ng for serum cfDNA. Then, the amplification mix was partitioned into ~20,000 wells loaded in a QuantStudio 3D Digital PCR Chip v2 and run on a ProFlex 2x Flat PCR System. The temperature profile for amplification was: An activation step of 10 min at 96 °C, followed by 40 cycles of 2 min at 60 °C, 30 s incubation at 98 °C, 2 min at 60 °C, and pause at 10 °C. The QuantStudio™ 3D analysisSuite™ was used to analyze the end-point fluorescence data to determine the proportion of templates, with and without a mutation, and to calculate the Mutation Allele Frequency (MAF). At least one negative and one positive control were added to each run.

### 2.6. TP53 Immunohistochemistry

FFPE tumor tissue sections of patients 1 and 2 were stained with polyclonal p53 antibodies clone DO-1 (1:200, Santa Cruz Biotechnogy, Heidelberg, Germany) and DO-7 (1:100, Dako, Santa Clara, USA) as described previously [18]. The DO-1 and DO-7 recognize overlapping epitopes. For patients 3–20, mouse monoclonal p53 antibody Bp53-11 (Ventana 769-2541, Ventana Medical Systems, Roche, Tuscon, AZ, USA) was used. Detection was performed using Ventana Benchmark Ultra detective with Ultraview Universal DAB detection kit (Ventana 760-500, Ventana Medical Systems, Roche, Tuscon, AZ, USA) and antigen retrieval Cell Conditioning Solution (CC1) (Ventana 950-124, Ventana Medical Systems, Roche, Tuscon, AZ, USA). The p53 expression was scored as previously described by Kobel et al. [19] and categorized into overexpression, complete absence, cytoplasmic, or wild-type.

### 2.7. Statistics

The study complied with reporting recommendations for tumor marker prognostic studies (REMARK) criteria [20]. Samples were called positive for TP53 non-synonymous mutations when the mutation frequency was above 1% when detected by Ampliseq panels or above 0.1% when detected by Oncomine or digital PCR assays. Statistical analyses were performed with Microsoft and Simple Interactive Statistical Analysis (SISA; http://www.quantitativeskills.com/sisa/index.htm). The analyses included student *t*-tests for continuous variables, Chi-square test for categorical variables. *p*-values were two-sided, and significance was defined at <0.05.

## 3. Results

### 3.1. TP53 Mutation and Protein Expression Analysis in Tumor Tissue

**Figure 1:**
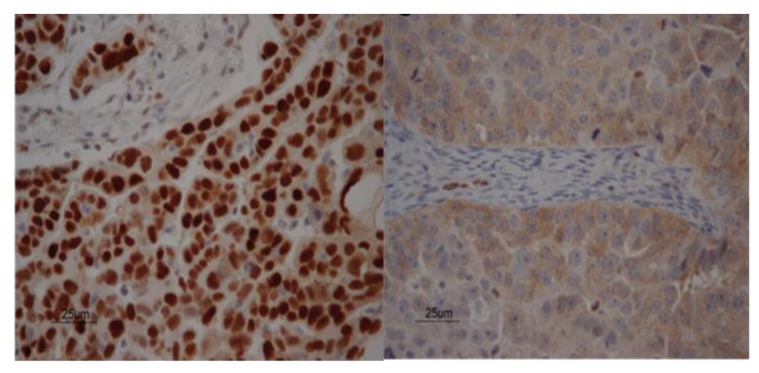
Tissue p53 staining. Two examples of immunohistochemical localization of p53 expression in patients with advanced stage ovarian cancer. Staining of sporadic nuclei with p53 antibody is seen in the stroma in both figures, acting as internal control. The p53 expression showed strong nuclear staining in patient 5 with a TP53 p.K132R mutation (left figure) and cytoplasmic staining in patient 6 without a *TP53* mutation (right figure).

First the *TP53* mutation status and protein expression were analyzed in tumor tissues. As *TP53* mutations in our archived serum not only might originate from tumor cells, but can also arise from clonal hematopoiesis, we first defined tumor-specific *TP53* mutations in tissue by NGS and immunohistochemistry. Missense *TP53* mutations and strong nuclear p53 protein expression were detected in all but three patients with no or synonymous mutations (Table 3, Figure 1). Only non-synonymous *TP53* mutations with (aberrant) nuclear staining in tumor tissue were then evaluated in serum cfDNA.

### 3.2. Serum cfDNA Yields at Diagnosis and Over Time

**Figure 2:**
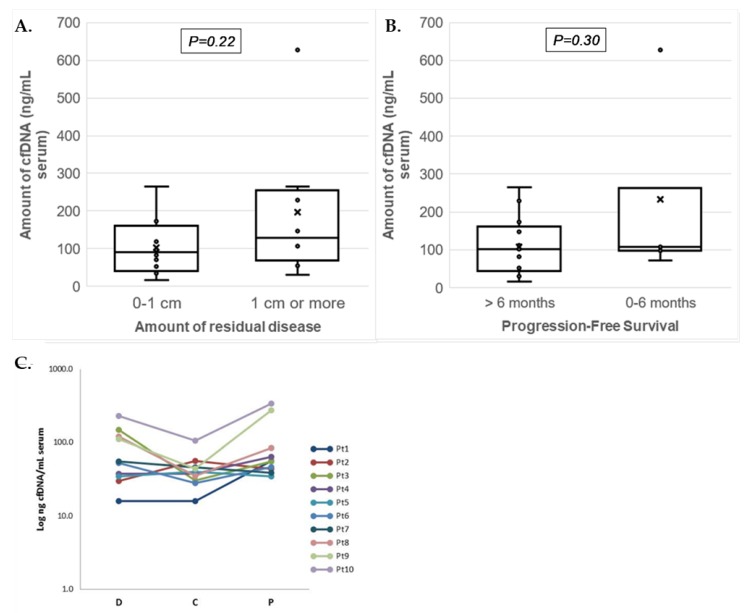
Serum cfDNA yields isolated from patients with advanced ovarian cancer. Boxplots presenting (**a**) amounts of cfDNA (ng/mL of serum) isolated at diagnosis for patients in relation to residual disease (RD; no or less than 1 cm vs. 1 cm or more) and (**b**) progression-free survival (PFS; ≤6 months and >6 months). The individual measurements are shown as dots, the mean by the cross (x), and median as horizontal line within the box. The cfDNA amounts between the groups of patients were not significantly different. (**c**) Graph showing the Log cfDNA concentration (ng/mL of serum) isolated from 10 patients at three different time points. Data points correspond to total cfDNA yields per mL serum for each patient (Pt) at the three different time points from serum collection: At diagnosis (D), after chemotherapy (C), and at disease progression (P).

The cfDNA yield per mL serum showed a range between 16 and 338 ng for workflow I and between 71 and 628 ng for workflow II (Table 3). Median cfDNA yields at diagnosis were comparable between non-optimal debulked patients (130ng/mL (N = 8)) and optimally debulked patients (90 ng/mL (N = 12); *p* = 0.218), and between patients with PFS shorter vs. longer than six months (median 107 (N = 5) and 102 ng/mL (N = 15); *p* = 0.30) (Figure 2A,B). The longitudinal serum cfDNA yields in workflow I were overall lower during chemotherapy (median: 39 ng/mL; *p* = 0.108) but similar at disease progression (median: 55 ng/mL; *p* = 0.627) when compared to cfDNA yields at diagnosis (median: 54 ng/mL) (Figure 2C).

### 3.3. Serum TP53 Mutation Detection at Diagnosis

In 9/17 patients (53%) with a TP53 missense mutation in tumor tissue, the mutation was also identified in serum cfDNA at diagnosis (Table 3; Supplementary Figure S1). These mutations were detected in 4/8 patients (50%) by Ampliseq NGS and in 4/6 patients (67%) by digital PCR for workflow I, and by Oncomine NGS in 6/9 patients (67%) for workflow II. dPCR was not performed at diagnosis for patients examined in the latest group due to the low amount of cfDNA available. Overall, *TP53* mutations in serum derived cfDNA at diagnosis were significantly more observed in patients with FIGO stage IV disease (*p* = 0.024; Table 4) but not related to other parameters including cfDNA yields or tumor tissue TP53 mutation frequencies (Table 4). Although not statistically significant, the number of patients with mutations detected in serum at diagnosis was higher in patients with non-optimal debulking surgery (4/6 patients (67%)) and disease progression within six months (3/4 patients (75%)) compared to patients with optimal debulking (5/11 patients (45%)) or progression after six months (6/13 patients (46%)). These exploratory findings should be verified in a larger set of patients.

### 3.4. Monitoring TP53 Mutations Over Time

**Figure 3:**
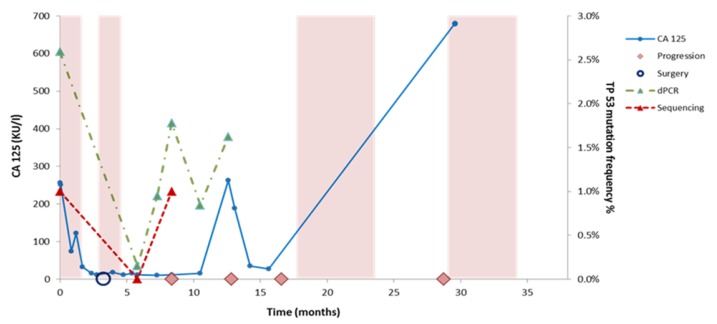
Monitoring cfDNA and CA125 levels over time in patient 5. Disease monitoring by CA125 levels and TP53 mutation (p.K132R) levels determined by NGS and digital PCR in patient 5. The colored boxes indicate time on treatment with chemotherapy. The graphs show the change in CA 125 (KU/l) levels and TP53 mutant allele frequencies (VAF %) in serial serum samples. The somatic mutations were measured using dPCR and Ion Torrent Sequencing. The timeline (in months) is indicated on the x-axis, the allele frequency of the identified mutations is represented on the right y-axis, while the CA 125 level is indicated on the left y-axis. The recolored boxes depict the times on treatment with chemotherapy. Surgery is indicated with a blue circle while clinical observed disease progression is depicted with a pink rhombus.

AmpliSeq NGS and dPCR were applied in workflow I to detect *TP53* mutations in sera taken during chemotherapy and at disease progression (Supplementary Figure S2). Both methods were unable to identify mutations during chemotherapy (not shown) but mutations were detected at disease progression in 3/8 patients (38%) by Ampliseq and in 5/8 patients (63%) by dPCR. The cfDNA *TP53* allele frequencies were lower at progression compared to baseline at diagnosis for all patients except patient 1 (Table 3). Interestingly, one patient with a *TP53* mutation at progression had this mutation not detected at diagnosis (Table 3). Further longitudinal monitoring of cfDNA was performed for patient 5 by the evaluation of CA125 levels and the dPCR monitoring of *TP53* p.K132R in cfDNA derived from additional sera. Low CA125 levels were measured between 5 and 10 months, whereas cfDNA levels increased upon disease progression (Figure 3).

### 3.5. cfDNA Workflow Comparison

Next to assay detection sensitivity, setup and costs are also important parameters to define an optimal workflow for monitoring *TP53* mutations in blood for (routine) cfDNA evaluation. First, Oncomine-NGS and dPCR (67%) were more sensitive assays than Ampliseq-NGS (50%) for detection of TP53 mutations in baseline cfDNA. The workflows (Table 2) differed in setup to evaluate TP53 only by NGS or combined with dPCR (workflow I) or multiple genes including TP53 by NGS only (workflow II). Secondly, the setup is important, since dPCR is only applicable when the patient-specific TP53 mutation is known from tumor tissue by Ampliseq-NGS or from cfDNA by Oncomine-NGS. Moreover, NGS enables the detection of additional mutations for *TP53* (workflow I) or for other genes as well (workflow II, Table 3), which might be acquired over time in longitudinal cfDNAs. Finally, current estimated costs differ between the two workflows and the applied setup. NGS only will be more expensive than tissue DNA NGS combined with dPCR of cfDNA (Table 2). For example, analyses of three longitudinal cfDNAs will cost €450–€750 by Ampliseq-NGS, €1050–€1350 by Oncomine NGS, whereas dPCR costs €460–€490 including €400 for designing the patient-specific TP53 mutation assay. The differences in costs between NGS and dPCR will increase much more when more longitudinal cfDNAs are evaluated. Workflow I with dPCR is only more cost-effective than NGS when at least 2 cfDNA samples are evaluated.

## 4. Discussion

In the current study, we investigated two NGS workflows to detect *TP53* mutations in tissue and serum cfDNA derived from patients with advanced stage serous ovarian cancer. Our aim was to establish the best method and to study whether *TP53* mutations can be used as a tumor biomarker in liquid biopsies. Patient-specific *TP53* missense mutations were identified by targeted NGS in tumor tissue and subsequently analyzed by NGS alone or in combination with dPCR in serum cfDNA taken at different timepoints.

The tumor suppressor TP53 gene is mainly mutated in exons 4–9, encoding for the DNA-binding domain of the protein [21]. It has been demonstrated that the aberrant protein is able to influence tumor progression toward migration, invasion, and metastasis in different tumor types [22,23,24,25,26]. In our study, 85% of patients harbored non-functional TP53 alterations in tumor tissue, which is in keeping with previous studies reporting TP53 mutations in more than 80% of HGSOC [27,28,29]. It has been shown by Kang et al. that HGSOC patients carrying a gain-of-function mutant p53 frequently develop resistance against platinum-based treatment as well as being more prone to develop distant metastasis [30]. Therefore, it seems that TP53 mutations might play a key role in the tumorigenesis of HGSOC.

Levels of cfDNA can vary widely in cancer patients as reported by Fleischhacker and Schmidt [31,32]. These authors reviewed 34 different studies and found that, although the cfDNA concentration in cancer patients is usually much higher than healthy controls, the amount varies widely and is often below 100 ng/mL. Our results are in line with these studies as the cfDNA yields measured at diagnosis were widely variable, although in 55% of cases, the yield was above 100 ng/mL. Yields of cfDNA in blood taken after chemotherapy and progression were, however, lower than 100 ng/mL in almost all cases.

Previous studies have demonstrated a correlation between tumor burden and cfDNA yields. It was shown that highly proliferative lesions release more cfDNA [33,34]. Other studies demonstrated that cfDNA amounts correlated with cancer survival rates [35], and showed its diagnostic value in different tumor types, such as lung cancer [36]. In contrast, we observed in our patient subset no significant differences in serum cfDNA amounts at diagnosis and over time in relation to clinical disease parameters.

Since cfDNA from tumor cells is released in the blood, the cfDNA mutational status may reflect the genetic characteristics of the primary or metastatic lesion [37,38]. Previous studies by Diaz and Bardelli demonstrated that mutations present in tumor tissue are virtually the same as those present in the cfDNA fraction [39,40]. Tumor-specific mutations in cfDNA could therefore act as prognostic and/or predictive biomarkers for cancer patients. In our study, we were able to detect the missense TP53 point mutation present in the primary tumor in cfDNA in 67% of patients. These results are in agreement with previous studies showing that point mutations in TP53 can be also measured in the serum DNA of patients with ovarian cancer [1]. In general, these results highlight the potential of cfDNA as diagnostic tool for ovarian cancer [5,12]. Our small cohort of HGSOC patients with FIGO stage IV disease showed a relation between levels of TP53 mutations detected in serum cfDNA at diagnosis but less with residual disease or disease progression.

Due to the small amounts of cfDNA and cfDNA available in blood, especially from retrospective archived serum, research is currently focused on the development of new strategies to quantify and characterize cfDNA. Sensitivity and specificity are the main challenges for detecting cancer-specific alterations in cfDNA. Recent advances in NGS [41,42,43] and PCR protocols have allowed the quantitative detection of mutations with a sensitivity below 0.001% [44]. Compared to conventional PCR, dPCR is a reliable method and easy to set up. Using this method, it is possible to quantify cfDNA without external references and with a higher sensitivity, precision, efficiency, and reproducibility [40]. In contrast, NGS allows identification of novel genetic or epigenetic mutations. Conventional NGS methods, however, are not as sensitive as dPCR methods and mutations could be missed, particularly when the total number of reads is low. Therefore, researchers have combined the use of NGS and dPCR protocols for liquid biopsy. When comparing the cost and time required for the different techniques, dPCR workflow is much cheaper because it needs less consumables and turnaround time to monitor a specific mutation in follow-up studies compared to NGS protocols. Moreover, dPCR enables accurate quantification of mutant DNA within vast amounts of wild-type DNA, i.e., low mutant allele frequencies (>0.1%), and is often used for the independent validation of NGS results.

Overall, Oncomine NGS in workflow II and dPCR in workflow I enabled detection of TP53 mutations below 1% allele frequencies and were more sensitive than the conventional Ampliseq NGS. This resulted in the detection of mutations in 67% of patients by dPCR and Oncomine NGS compared to only 50% of patients by Ampliseq NGS. Moreover, to trace TP53 mutations for disease monitoring in multiple longitudinal serum derived cfDNA, we showed that dPCR is better compared to Oncomine NGS, due to lower amounts for cfDNA needed and lower overall costs. However, a proper direct comparison of mutation detection sensitivity of each platform was not possible due to the limitations of our study design. Summarizing, study restrictions were the limited amounts of archived serum available (>1mL), low cfDNA yields, and different (recommended) cfDNA input amounts for each NGS platform. To accomplish an accurate comparison of detection sensitivities, equal cfDNA input amounts should be evaluated by both NGS platforms in future studies. We also compared the cost-effectiveness of the two workflows, which is of relevance when introducing the methodology to routine diagnostic of HGSOC.

To date, several clinical studies have tried to link TP53 mutations with patient survival or the development of chemoresistance [45]. However, the conclusions of these studies are often contradictory due to the unselective classification of all TP53 mutations and the single use of immunohistochemistry to determine the TP53 mutational status. Recently, it has been proposed that TP53 mutations could be used as biomarkers to predict patient response to chemotherapy. In line with these studies, we were unable to identify TP53 mutations at chemotherapy but detected these mutations at disease progression for 5/6 patients. Parkinson et al. [14] reported that cfDNA TP53 mutant allele fraction and volumetric measurements were correlated in HGSOC patients, particularly in a subset of patients without ascites. Moreover, almost all subjects with disease volume larger than 32 cm3 showed higher cfDNA copies. A rapid response to chemotherapy was more closely related to cfDNA than to CA-125. These results strongly suggest that cfDNA has the potential to be a highly specific early molecular response marker in HGSOC [14,40].

## 5. Conclusions

In conclusion, detection of tumor-specific *TP53* missense mutations in minute amounts of archived serum-derived cfDNA from HGSOC patients is enabled by dPCR or NGS. Our exploratory finding that *TP53* mutations present at diagnosis became undetectable in cfDNA after chemotherapy but re-appeared at disease progression highlights the potential role of *TP53* missense mutations as a biomarker for clinical disease monitoring in ovarian cancer. However, detection sensitivities of NGS platforms need to be validated further in a larger study.

## Figures and Tables

**Table 1:** Study design and clinical characteristics.

	Study Design Details			Clinical Characteristics			
Patient	Workflow	Sample Subset	Age	FIGO Stage	Debulking Surgery *	Residual Disease	Progression-Free Survival (PFS)
1	**NGS & dPCR:** *TP53* only	Tissue & longitudinal cfDNA	43	IV	PDS	0–1 cm	>6 months
2			37	IIIC	PDS	1 cm or more	>6 months
3			69	IIIC	PDS	1 cm or more	>6 months
4			56	IV	PDS	0–1 cm	>6 months
5			73	IIIC	IDS	0–1 cm	>6 months
6 **			53	IIIC	PDS	0–1 cm	>6 months
7			58	IV	PDS	1 cm or more	>6 months
8			58	IIIC	PDS	0–1 cm	>6 months
9			72	IIIC	IDS	1 cm or more	>6 months
10 **			75	IV	IDS	1 cm or more	>6 months
11	**NGS only:** Multigene panels including *TP53*	Tissue & baseline cfDNA	47	IV	IDS	0–1 cm	>6 months
12			47	IIIC	IDS	0–1 cm	>6 months
13			57	IIIC	IDS	0–1 cm	>6 months
14			49	IC	PDS	0–1 cm	>6 months
15			61	IV	IDS	0–1 cm	>6 months
16			63	IIIC	IDS	0–1 cm	0–6 months
17			45	IV	IDS	0–1 cm	0–6 months
18 **			65	IV	NA *	1 cm or more	0–6 months
19			47	IV	NA *	1 cm or more	0–6 months
20			72	IV	IDS	1 cm or more	0–6 months

* PDS = primary Debulking surgery, IDS = interval Debulking surgery, NA = not available. ** Patients were excluded from cfDNA analysis because their tissue had no *TP53* missense mutations.

**Table 2:** Cell-free DNA (cfDNA) workflow details.

	Worflow I: NGS & dPCR—*TP53* only		Worflow II: NGS only—Multigene Hotspot Panels Including *TP53*	
**Samples**	Tissue DNA	Longitudinal cfDNA	Tissue DNA	Baseline cfDNA only
**NGS panel names & details**	Ampliseq TP53 community panel	Ampliseq TP53 community panel	Ampliseq Customized Diagnostic panel	Oncomine breast cfDNA assay with molecular barcoding
**Panel details**	24 amplicons, 2550 bp		328 amplicons, 35793 bp, 41 genes	26 amplicons, 4420 bp, 10 genes
**Input amount**	10 ng	1.5–3.3 ng	10 ng	15–20 ng
**Mean reads depth coverage (range)**	1725 reads/amplicon (943–3584 reads)	1851 reads/amplicon (843–3776 reads)	1166 reads/amplicon (430–1796 reads)	32902 reads/amplicon (5786–59976 reads)
**Estimated costs per sample for:**				
NGS	€150–€250	€150–€250	€250–€350	€350–€450
dPCR (€400 per mutation assay)	€20–€30	€20–€30	NA	NA
**Workflow cfDNA NGS vs. dPCR total costs for:**				
Baseline cfDNA only		€150–€250 vs. €420–€430		€350–€450 vs. €420–€430
cfDNA at baseline & progression		€300–€500 vs. €440–€460		€700–€900 vs. €440–€460
3 longitudinal cfDNAs		€450–€750 vs. €460–€490		€1050–€1350 vs. €460–€490
5 longitudinal cfDNAs		€750–€1250 vs. €500–€550		€1750–€2250 vs. €500–€550

**Table 3:** Tumor tissue and serum characteristics.

					cfDNA Yields per mL Serum (ng/mL)				cfDNA MAF (NGS)		cfDNA MAF (dPCR)			
Subset	Patient	Identified *TP53* Mutation in Tissue	MAF Tissue	TP53 IHC	D	C	P	Excludedin cfDNA Analysis	D	P	D	P	Additional Mutation(s)	MAF
**I. NGS & dPCR:** *TP53* only	1	p.Y163C	57%	nuclear ++	17	16	56		0.0%	0.0%	0.0%	0.8%		
	2	p.C275Y	27%	nuclear ++	30	56	44		1.0%	0.0%	5.3%	0.1%		
	3	p.P151R	73%	nuclear ++	148	30	55		0.0%	6.0%	NE	NE		
	4	p.R282W	78%	nuclear ++	37	39	64		30.0%	0.0%	31.9%	0.4%		
	5	p.K132R	21%	nuclear ++	35	40	35		1.0%	1.0%	2.6%	1.8%		
	6	No mutation	-	cytoplasmatic	52	28	46	no mutation	-	-	-	-		
	7	p.Y163C	64%	nuclear ++	56	46	39		2.0%	1.0%	6.2%	2.9%		
	8	p.C275Y	7%	nuclear ++	119	35	84		0.0%	0.0%	0.0%	0.0%		
	9	p.C277F	58%	nuclear ++	111	44	275		0.0%	0.0%	NE	NE		
	10	p.N131N	3%	cytoplasmatic	229	106	338	synonymous	-		NE	NE		
**II. NGS only:** Multigene hotspot panels including *TP53*	11	p.E286G	81%	nuclear ++	174				1.9%					
	12	p.Y205D	51%	nuclear ++	174				0.0%					
	13	p.F134V	75%	nuclear ++	102				0.0%				*TP53* p.T253I	0.1%
	14	p.K132R	79%	nuclear ++	265				0.0%					
	15	p.L194R	88%	nuclear ++	83				0.3%					
	16	p.Y220C	65%	nuclear ++	98				0,0%				*ESR1* p.R394S	0.3%
	17	p.C176W	56%	nuclear ++	71				25.6%					
	18	Unknown	-	N/A	628			unknown	-					
	19	p.E258G	85%	nuclear ++	264				0.6%					
	20	p.P278S	60%	nuclear ++	107				0.5%				*PIK3CA* p.H1047R	1.3%

IHC = immunohistochemistry, D = at diagnosis, C = after chemotherapy, P = at disease progression, NGS = next generation sequencing, dPCR = digital PCR, MAF = mutant allele frequency, unknown = mutation was unknown since no tissue was available, N/A = not available, ++ = high TP53 levels nuclear staining.

**Table 4:** Comparison clinicopathological characteristics of high-grade serous ovarian carcinomas (HGSOC) patients with and without serum tumor-specific *TP53* mutation at diagnosis.

HGSOC Patients Serum cfDNA at Diagnosis:			
	Without Tumor-Cpecific *TP53* Mutation	With Tumor-Specific *TP53* Mutation	*p*-Value
**Number of patients**	8	9	
**Average TP53 mutation allele frequency (MAF in %):**			
in tumor tissue	58%	62%	0.819
in cfDNA	0%	7%	0.115
**NGS workflow (N):**			
I	4	4	0.819
II	4	5	
**Age at diagnosis (average):**	57	55	0.703
**FIGO Stage (N):**			
IC	1	0	0.024
IIIC	6	2	
IV	1	7	
**Debulking surgery (N):**			
PDS	4	3	0.614
IDS	4	5	
**Residual Disease (N):**			
0–1 cm (optimal debulking)	6	5	0.402
1 cm or more (non-optimal debulking)	2	5	
**Progression-Free Survival (PFS):**			
0–6 months (n)	1	3	0.312
>6 months (n)	7	6	
**average cfDNA yield (ng/mL serum):**			
at diagnosis	129	95	0.363
after chemotherapy	31	45	0.106
at progression	117	45	0.268
**TP53 mutation at progression measured by dPCR (N):**			
Yes	1	4	0.079
No	1	0	
**Other gene mutations detected in cfDNA at diagnosis:**			
*PIK3CA* p.H1047R		1.30%	
*ESR1* p.R394S	0.30%		
*TP53* p.T253I *	0.10%		

PDS = primary Debulking surgery, IDS = interval Debulking surgery, NGS = next generation sequencing, dPCR = digital PCR, N = number of patients, * = *TP53* p.T253I was identified in serum but not in tumor tissue.

## Supplementary Materials

The following are available online at https://www.mdpi.com/2218-273X/10/3/415/s1, Table S1: Digital PCR SNP genotyping assays used for subset I, Table S2: Digital PCR primers and probe sequences, Figure S1: *TP53* mutation analysis by digital PCR in patient serum.
