# Supplementary material for: TP53 Mutations in Serum Circulating Cell-Free Tumor DNA As Longitudinal Biomarker for High-Grade Serous Ovarian Cancer
Source: Biomolecules. 2020 Mar 7;10(3):415. doi: 10.3390/biom10030415 (PMC7175353; doi:10.3390/biom10030415)
Supplement: Supplementary file 1 [file biomolecules-10-00415-s001.pdf]

Legends supplementary tables and figures

Supplementary table S1. Digital PCR SNP genotyping assays used for subset I.

Assay ID	Assay Name	Gene	Cosmic ID	Amino acid change	Nucleotide change
AHKA3Z2	TP53_K132R	*TP53*	11582	p.K132R	c.395A>G
AHLJ16A	TP53_P151R	*TP53*	44003	p.P151R	c.452C>G
AHMS0CI	TP53_Y163C	*TP53*	10808	p.Y163C	c.488A>G
AHI15TU	TP53_C275Y	*TP53*	10893	p.C275Y	c.824G>A
AH705J0	TP53_C277F	*TP53*	10749	p.C277F	c.830G>T
AHRSSQL	TP53_R282W	*TP53*	10704	p.R282W	c.844C>T

Supplementary Table S2. Custom Primer and Probe sequences used for digital PCR.

Assay Name	Sequence
TP53_K132R	Fw: 5′-GCAGGTCTTGGCCAGTTG -3′
	Rev: 5′-GTCTCCTTCCTCTTCCTACAGTACT -3′
	VIC Probe: 5′-CCCTCAACAAGATGTT -3′
	FAM Probe: 5′- CCCTCAACAGGATGTT-3′
TP53_P151R	Fw: 5′-TGTGCTGTGACTGCTTGTAGATG -3′
	Rev: 5′-TGTGCAGCTGTGGGTTGAT -3′
	VIC Probe: 5′- TCCACACCCCCGCCC-3′
	FAM Probe: 5′-CACACGCCCGCCC -3′
TP53_Y163C	Fw: 5′-CCTCCGTCATGTGCTGTGA -3′
	Rev: 5′-GCAGCTGTGGGTTGATTCCA -3′
	VIC Probe: 5′-CATGGCCATCTACAAGC -3′
	FAM Probe: 5′-ATGGCCATCTGCAAGC -3′
TP53_C275Y	Fw: 5′- CTGTGCGCCGGTCTCT-3′
	Rev: 5′-TGGGACGGAACAGCTTTGAG -3′
	VIC Probe: 5′-TGCGTGTTTGTGCCTG-3′
	FAM Probe: 5′-TGCGTGTTTATGCCTG -3′
TP53_C277F	Fw: 5′-CTGTGCGCCGGTCTCT-3′
	Rev: 5′-TGGGACGGAACAGCTTTGAG-3′
	VIC Probe: 5′-TGTTTGTGCCTGTCCTGG-3′
	FAM Probe: 5′-TGTTTGTGCCTTTCCTGG-3′

Fw: Forward primer, Rev: Reverse Primer; VIC Probe: VIC Taqman probe, FAM Probe: FAM Taqman probe.

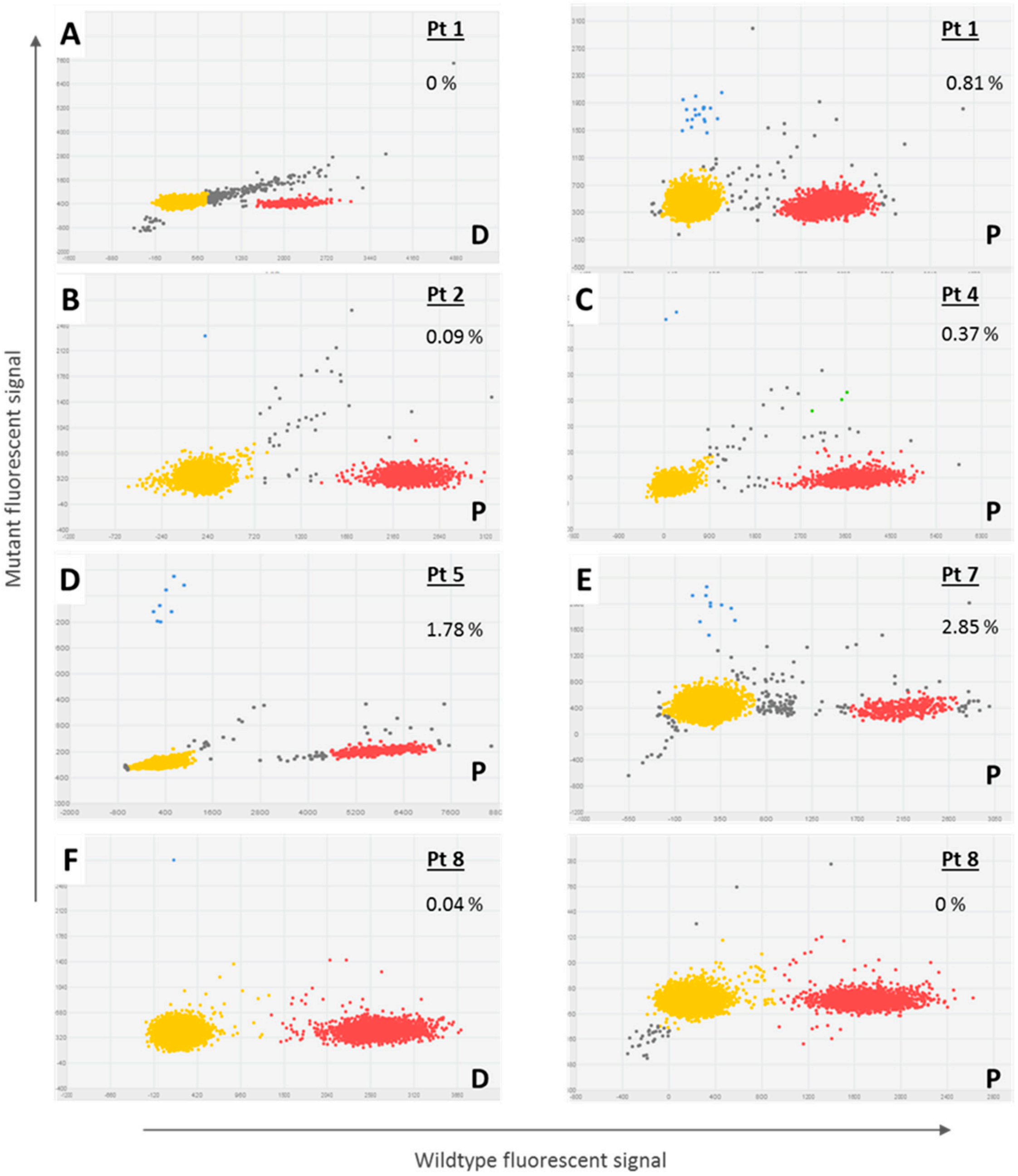

Supplemental Figure S1. *TP53* mutation analysis by digital PCR in patient serum. *TP53* mutation analysis by digital PCR in patient serum. The figure shows dot plots indicating the presence of wildtype (WT) and mutant (MT) copies in cfDNA of **A)** patient 1 analyzed for TP53_pY163C. **B)** patient 2 analyzed for TP53_pC275Y, **C)** patient 4 analyzed for TP53_pR282W, **D)** patient 5 analyzed for TP53_pK132R**, E)** patient 7 analyzed for TP53_pY163C and **F)** patient 8 analyzed for TP53_pC275Y at diagnosis and/or progressive disease. Blue: wells containing mutant copies, Red: wells containing wildtype copies, Green: wells that contain both wildtype and mutant copies, Yellow empty wells, Grey: undetermined wells, VAF: Variant Allele Frequency. D: diagnosis, P: progression disease.

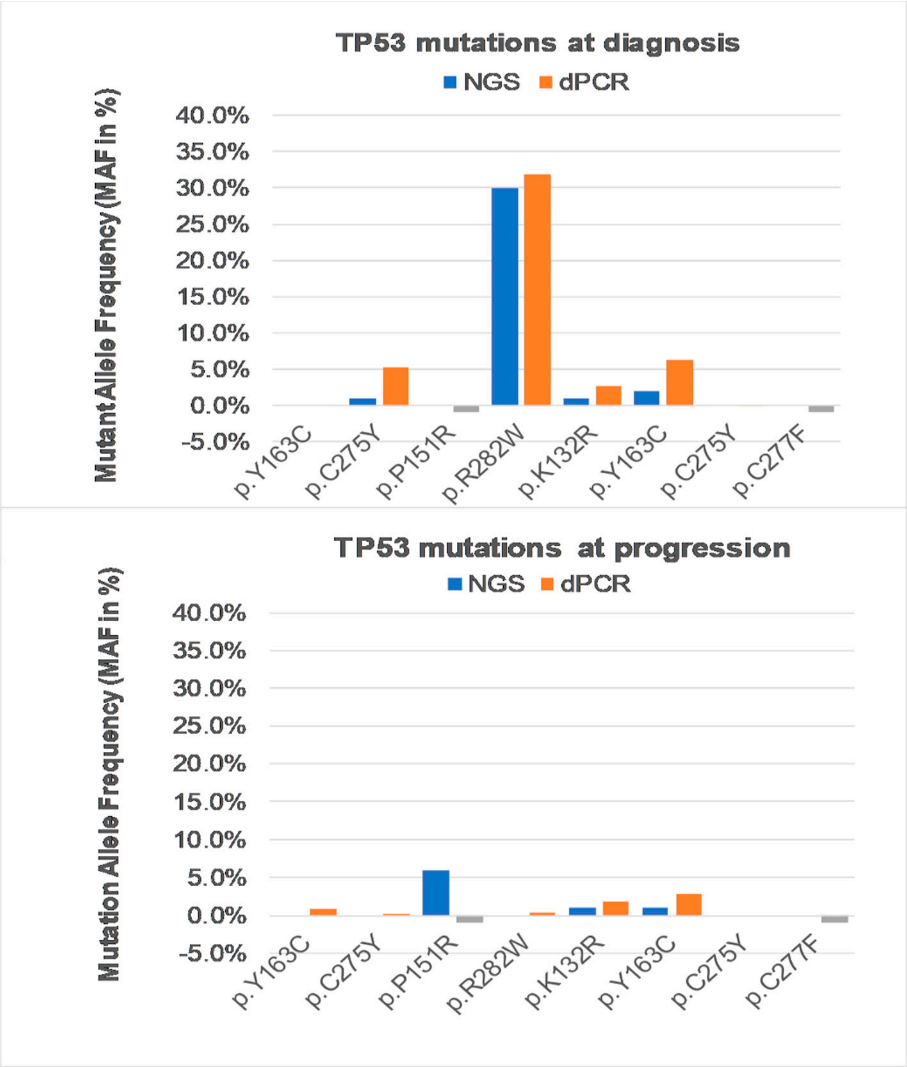

Supplemental Figure S2. Tumor-specific *TP53* measured by NGS and digital PCR in cfDNATumor-specific *TP53* mutations were measured by Ampliseq NGS (blue bars) and digital PCR (dPCR, orange bars) in cfDNA of archived serum taken at diagnosis, during chemotherapy and at progression. Both NGS and dPCR did not detect any *TP53* mutation in serum during treatment. The TP53_ pP151R and TP53_pC277F mutation were detected by NGS, but dPCR failed (grey bars). The dPCR detected mutations at higher mutation allele frequency than NG and more often at progression (TP53_pY163C, TP53_pC275Y, TP53_pR282W). MAF= Mutation allele frequency; NGS= next generation sequencing, dPCR= digital PCR.
